# Multi-Target Joint Detection and Estimation Error Bound for the Sensor with Clutter and Missed Detection

**DOI:** 10.3390/s16020169

**Published:** 2016-01-28

**Authors:** Feng Lian, Guang-Hua Zhang, Zhan-Sheng Duan, Chong-Zhao Han

**Affiliations:** Ministry of Education Key Laboratory for Intelligent Networks and Network Security (MOE KLINNS), College of Electronics and Information Engineering, Xi’an Jiaotong University, Xi’an 710049, China; MichaelZgh@stu.xjtu.edu.cn (G.-H.Z.); zsduan@xjtu.edu.cn (Z.-S.D.); czhan@xjtu.edu.cn (C.-Z.H.)

**Keywords:** performance evaluation, error bound, multi-target tracking, joint detection and estimation, random finite set

## Abstract

The error bound is a typical measure of the limiting performance of all filters for the given sensor measurement setting. This is of practical importance in guiding the design and management of sensors to improve target tracking performance. Within the random finite set (RFS) framework, an error bound for joint detection and estimation (JDE) of multiple targets using a single sensor with clutter and missed detection is developed by using multi-Bernoulli or Poisson approximation to multi-target Bayes recursion. Here, JDE refers to jointly estimating the number and states of targets from a sequence of sensor measurements. In order to obtain the results of this paper, all detectors and estimators are restricted to maximum *a posteriori* (MAP) detectors and unbiased estimators, and the second-order optimal sub-pattern assignment (OSPA) distance is used to measure the error metric between the true and estimated state sets. The simulation results show that clutter density and detection probability have significant impact on the error bound, and the effectiveness of the proposed bound is verified by indicating the performance limitations of the single-sensor probability hypothesis density (PHD) and cardinalized PHD (CPHD) filters for various clutter densities and detection probabilities.

## 1. Introduction

The problem of joint detection and estimation (JDE) of multiple targets arises from many applications in surveillance and defense [1], where the number of targets is unknown and the sensor may receive measurements generated randomly from either targets or clutters. There is no information about which are the measurements of interest or which are the clutters. The aim of multi-target JDE is to determine the number of targets and to estimate their states if exist using prior information, as well as a sequence of the sensor measurements. In recent years, multi-target JDE has attracted extensive attention, and many approaches for it have been proposed [2,3,4,5,6,7,8,9,10].

Obviously, it is very necessary to find an error (lower) bound to assess the achievable performance of the multi-target JDE algorithms for the given sensor measurements. It is well known that Tichavsky *et al.* [11] proposed a recursive posterior Cramér-Rao lower bound (CRLB) for evaluating the performance of nonlinear filters when a target was asserted and observed by a sensor. Then, the CRLB was extended to the cases in which clutter or missed detection was present in the sensor [12,13,14,15]. Nevertheless, these CRLBs [12,13,14,15] could barely be applied to such a JDE problem, since CRLB only considers the estimation error of a target state, but not the detection error of the target number (or existence/non-existence of a target). Within random finite set (RFS) [2,4] framework, Rezaeian and Vo [16] derived the static error bounds for JDE of a single target observed by a single sensor with clutter and missed detection. Tong *et al*. presented a recursive form of a single-sensor single-target error bound based on CRLB when only missed detection, but not clutter, exists [17] and then extended the result of [17] to the single-sensor multi-target case with the more rigorous restriction that neither clutter nor missed detection exists [18]. Note that the bounds in [17,18] actually do not include the detection error generated by the uncertainty of target number, since the target number can be completely determined by the measurement number by restricting the sensor observation model to the one in [17,18].

This paper proposes an RFS-based single-sensor multi-target JDE error bound when clutter and missed detection may simultaneously exist in the sensor. In order to obtain the results of this paper, the multi-target Bayes recursion is approximated as a multi-Bernoulli process [2] or a Poisson process [2], and all detectors and estimators are restricted to maximum *a posterior* (MAP) detectors and unbiased estimators. Since the JDE error is the average distance between true and estimated state sets, the second-order optimal sub-pattern assignment (OSPA) distance [19] rather than the Euclidean distance is used as the error metric. Finally, the simulation results show that clutter density and detection probability have significant impacts on the proposed bound, and the effectiveness of the proposed bound is verified by indicating the performance limitations of the single-sensor probability hypothesis density (PHD) [4] and cardinalized PHD (CPHD) [5] filters for various clutter densities and detection probabilities.

The rest of the paper is organized as follows. Section 2 presents the background for deriving our results. In Section 3, we derive the proposed bound by using multi-Bernoulli or Poisson approximation. A numerical example is presented in Section 4. The conclusions and future work are given in Section 5. Relevant mathematical proofs are provided in Appendix A and Appendix B.

## 2. Background

- Set integral: For any real-valued function φ(X) of a finite-set variable *X*, its set integral is [4]:
  (1) $$\int \varphi (X)\delta X=\sum_{n=0}^{\infty }\frac{1}{n!}\int_{X^{n}}\varphi \left(X^{n}\right)dx^{\left(1\right)}\cdots dx^{\left(n\right)}=\varphi \left(\emptyset \right)+\sum_{n=1}^{\infty }\frac{1}{n!}\int_{X^{n}}\varphi \left(X^{n}\right)dx^{\left(1\right)}\cdots dx^{\left(n\right)}$$
  where $X^{n}={\left\{x^{\left(i\right)}\right\}}_{i=1}^{n}\subseteq X^{n}$ denotes a *n*-points set (that is, the cardinality of the set $X^{n}$ is *n*) and $X^{n}$ denotes the space of $X^{n}$. In this paper, we note $X^{0}=\emptyset$.
- Multi-Bernoulli RFS: A multi-Bernoulli RFS *X* is a union of *M* independent Bernoulli RFSs $X^{\left(i\right)}$, $X={⋃}_{i=1}^{M}X^{\left(i\right)}$. Its density is completely described by parameter $\Upsilon ={\left\{\left(r^{\left(i\right)},p^{\left(i\right)}\right)\right\}}_{i=1}^{M}$ as [6]:
  (2) $$f\left(X\right)=\pi \left(\emptyset \right)\sum_{1\leq j_{1}\neq \cdot \cdot \cdot \neq j_{\left|X\right|}\leq M}\prod_{i=1}^{\left|X\right|}\frac{r^{\left(j_{i}\right)}}{1-r^{\left(j_{i}\right)}}p^{\left(j_{i}\right)}\left(x^{\left(i\right)}\right),\;\;\text{with}\;\;\pi \left(\emptyset \right)=\prod_{i=1}^{M}\left(1-r^{\left(i\right)}\right)$$
  where |·| denotes the cardinality of a set, $r^{\left(i\right)}\in \left(0,1\right)$ denotes the probability of $X^{\left(i\right)}\neq \emptyset$ and $p^{\left(i\right)}\left(x^{\left(i\right)}\right)$ denotes the density of $x^{\left(i\right)}$.
- Poisson RFS: An RFS *X* is Poisson if its density f(X) is:
  (3) $$f\left(X\right)=e^{-\eta }\prod_{x\in X}\upsilon (x),\;\;\text{with}\;\;\eta =\int \upsilon (x)dx\;\;\text{and}\;\;\upsilon \left(x\right)=\eta f\left(x\right)$$
  where υ(x) denotes the intensity function of the Poisson RFS *X*, *η* is the average number of elements in *X* and f(x) is the density of single element x∈X.
- Second-order OSPA distance: The OSPA distance of order p=2 between set *X* and its estimate $\hat{X}$ is [19]:
  (4) $$d^{2}\left(X,\hat{X}\right)=\left\{\begin{array}{c} \begin{array}{cc} 0, & |\hat{X}|=|X|=0 \end{array}\\ \begin{array}{cc} \frac{\min_{\tau \in \Pi_{\max(|\hat{X}|,|X|)}}\sum_{t=1}^{\min(|\hat{X}|,|X|)}\min\left(c^{2},{∥x^{\left(t\right)}-{\hat{x}}^{\left(\tau (t)\right)}∥}_{2}^{2}\right)+c^{2}\left||\hat{X}\left|-|X\right|\right|}{\max(|\hat{X}|,|X|)}, & |\hat{X}|+|X|>0 \end{array} \end{array}\right.$$
  where $\Pi_{n}$ denotes the set of permutations on {1,2,…,n}, c>0 denotes the cut-off parameter, max(·) or min(·) denotes the maximization or minimization operation and $\left||\cdot \right|{|}_{2}$ denotes the two-norm. The OSPA metric is comprised of two components, each separately accounting for “localization” and “cardinality” errors between two sets. The localization error arises from the estimates paired with the nearest truths, while the cardinality error arises from the unpaired estimates. Schuhmacher *et al.* [19] have proven that the OSPA distance with p∈[1,∞) and c>0 is indeed a metric, so it can be used as a principled performance measure.
- Information inequality and CRLB: Given a joint probability density f(x,z) on X×Z, under regularity conditions and the existence of $\partial^{2}\log f\left(x,z\right)/\partial x_{i}\partial x_{j}\;$, the information inequality states that [20,21]:
  (5) $$\int_{Z}\int_{X}f\left(x,z\right){\left(x_{l}-{\hat{x}}_{l}\left(z\right)\right)}^{2}dxdz\geq {\left(\frac{\partial }{\partial x_{l}}E_{f}\left[{\hat{x}}_{l}\left(z\right)\right]\right)}^{2}\cdot {\left[J^{-1}\right]}_{l,l}$$
  where $\hat{x}\left(z\right)$ denotes an estimate of *L* dimensional vector x based on z, $x_{l}$ and ${\hat{x}}_{l}\left(z\right)$ are, respectively, the *l*-th components of x and $\hat{x}\left(z\right)$, l=1,…,L, the notation $E_{f}$ means the expectation with respect to density *f* and *J* is known as the L×L Fisher information matrix:
  (6) $$\begin{array}{cc} {\left[J\right]}_{i,j}=-E_{f}\left[\frac{\partial^{2}\log f\left(x,z\right)}{\partial x_{i}\partial x_{j}}\right]=-\int_{Z}\int_{X}f\left(x,z\right)\frac{\partial^{2}\log f\left(x,z\right)}{\partial x_{i}\partial x_{j}}dxdz, & i,j=1,2\ldots ,L \end{array}$$
  where ${\left[J\right]}_{i,j}$ denotes the element on the *i*-th row and *j*-th column of matrix *J*.
  For the particular case in which the estimator $\hat{x}\left(z\right)$ is unbiased (that is, $E_{f}\left[\hat{x}\left(z\right)\right]=x$), the information inequality of Equation (5) reduces to:
  (7) $$\int_{Z}\int_{X}f\left(x,z\right){\left(x_{l}-{\hat{x}}_{l}\left(z\right)\right)}^{2}dxdz\geq {\left[J^{-1}\right]}_{l,l}$$
  which is a result known as the CRLB. The Fisher information matrix *J* in Equation (7) is also computed by Equation (6).
  Note that the ordinary information inequality of Equation (5) holds without the unbiasedness requirement on the estimator $\hat{x}\left(z\right)$. However, unbiasedness is critical in the CRLB of Equation (7).
  Explanation: In the current set up of this paper, our attention is restricted to the unbiased estimator of multi-target states. Our future work will study the extension of the proposed bound to the biased estimator by using the ordinary information inequality of Equation (5).
  Moreover, Equation (5) or Equation (7) is satisfied with equality depending on a very restricted condition. In [21], Poor concludes that, within regularity, the information lower bound is achieved (that is, the “=” in Equation (5) or Equation (7) holds) by $\hat{x}\left(z\right)$ if and only if $\hat{x}\left(z\right)$ is in a one-parameter exponential family (e.g., the linear Gaussian models for target dynamics and sensor observation described in [11] for achieving the CRLB). More details about this can be found in [21].
- RFS-based multi-target dynamics and sensor observation models: Let $x_{k}\in X_{k}$ denote the state vector of a target and $X_{k}$ the set of multi-target states at time *k*, where $X_{k}$ is the state space of a target. The multi-target dynamics is modeled by:
  (8) $$X_{k}=\left[\underset{x_{k-1}\in X_{k-1}}{\cup }\Psi_{k|k-1}\left(x_{k-1}\right)\right]\cup \Gamma_{k}$$
  where $\Psi_{k|k-1}\left(x_{k-1}\right)$ is the set evolved from the previous state $x_{k-1}$, $\Psi_{k|k-1}\left(x_{k-1}\right)=\left\{x_{k}\right\}$ with surviving probability $p_{S,k}\left(x_{k-1}\right)$ and transition density $f_{k|k-1}\left(\left.x_{k}\right|x_{k-1}\right)$, otherwise $\Psi_{k|k-1}\left(x_{k-1}\right)=\emptyset$ with probability $1-p_{S,k}\left(x_{k-1}\right)$; $\Gamma_{k}$ is the set of spontaneous births.
  Let $z_{k}\in Z_{k}$ denote a measurement vector and $Z_{k}$ the set of measurements received by a sensor at time *k*, where $Z_{k}$ is the sensor measurement space. The single-sensor multi-target observation is modeled by:
  (9) $$Z_{k}=\left[\underset{x_{k}\in X_{k}}{\cup }\Theta_{k}\left(x_{k}\right)\right]\cup K_{k}$$
  where $\Theta_{k}\left(x_{k}\right)$ is the measurement set originated from state $x_{k}$, $\Theta_{k}\left(x_{k}\right)=\left\{z_{k}\right\}$ with sensor detection probability $p_{D,k}\left(x_{k}\right)$ and likelihood $g_{k}\left(\left.z_{k}\right|x_{k}\right)$, otherwise $\Theta_{k}\left(x_{k}\right)=\emptyset$ with probability $1-p_{D,k}\left(x_{k}\right)$; $K_{k}$ is the clutter set, which is modeled as a Poisson RFS with density:
  (10) $$f_{c,k}\left(K_{k}\right)=e^{-\lambda_{k}}\prod_{z_{k}\in K_{k}}\kappa_{k}\left(z_{k}\right),\;\;\text{with}\;\;\lambda_{k}=\int \kappa_{k}\left(z_{k}\right)dz_{k}\;\;\text{and}\;\;\kappa_{k}\left(z_{k}\right)=\lambda_{k}f_{c,k}\left(z_{k}\right)$$
  where $\kappa_{k}\left(z_{k}\right)$ is the clutter intensity, $\lambda_{k}$ is the average clutter number and $f_{c,k}\left(z_{k}\right)$ is the density of a clutter.
  The transition model in Equation (8) jointly incorporates motion, birth and death for multiple targets, while the sensor observation model in Equation (9) jointly accounts for detection uncertainty and clutter. Assume that the RFSs constituting the unions in Equations (8) and (9) are mutually independent. The multi-target JDE at time *k* is to derive the estimated state set ${\hat{X}}_{k}\left(Z_{1:k}\right)$ using the collection $Z_{1:k}=Z_{1},\ldots ,Z_{k}$ of all sensor observations up to time *k*. The paper aims to derive a performance limit to multi-target joint detectors-estimators for the observation of a single sensor with clutter and missed detection. The performance limit is measured by the bound of the average error between $X_{k}$ and ${\hat{X}}_{k}\left(Z_{1:k}\right)$.

## 3. Single-Sensor Multi-Target JDE Error Bounds Using Multi-Bernoulli or Poisson Approximation

At time *k*, the RFS-based mean square error (MSE) between $X_{k}$ and ${\hat{X}}_{k}\left(Z_{1:k}\right)$ is defined as:

(11) $$\begin{array}{cc} \sigma_{k}^{2} & =E\left[e_{k}^{2}\left(X_{k},{\hat{X}}_{k}\left(Z_{1:k}\right)\right)\right]\\ & =\int \int f_{k}\left(\left.X_{k},Z_{k}\right|Z_{1:k-1}\right)e_{k}^{2}\left(X_{k},{\hat{X}}_{k}\left(Z_{1:k}\right)\right)\delta X_{k}\delta Z_{k}\\ & =\int \int \gamma_{k}\left(\left.Z_{k}\right|X_{k}\right)f_{k|k-1}\left(\left.X_{k}\right|Z_{1:k-1}\right)e_{k}^{2}\left(X_{k},{\hat{X}}_{k}\left(Z_{1:k}\right)\right)\delta X_{k}\delta Z_{k} \end{array}$$

where $e_{k}\left(X_{k},{\hat{X}}_{k}\left(Z_{1:k}\right)\right)$ denotes the error metric between $X_{k}$ and ${\hat{X}}_{k}\left(Z_{1:k}\right)$, which is defined by the second-order OSPA distance in (4), $f_{k}\left(\left.X_{k},Z_{k}\right|Z_{1:k-1}\right)$ denotes the density of $\left(X_{k},Z_{k}\right)$ given $Z_{1:k-1}$ and $\gamma_{k}\left(\left.Z_{k}\right|X_{k}\right)=f_{k}\left(\left.Z_{k}\right|X_{k}\right)$ denotes the likelihood for the total sensor measurement process.

At time *k*, given multi-target state set $X_{k}^{n}$ and sensor measurement set $Z_{k}^{m}$, all association hypotheses can be represented as a function from target index set {1,…,n} to sensor measurement index set {0,1,…,m} [2]. Defining that:

(12) $$\begin{array}{cc} \theta_{n,m}: & \left\{1,\ldots ,n\right\}\to \left\{0,1,\ldots ,m\right\} \end{array}$$

denotes the association hypothesis function with clutter and missed detection. That is, the *t*-th target $x_{k}^{\left(t\right)}$ with $\theta_{n,m}\left(t\right)=0$ generates no detection, while target $x_{k}^{\left(t\right)}$ with $\theta_{n,m}\left(t\right)>0$ generates a sensor measurement $z_{k}^{\left(\theta_{n,m}\left(t\right)\right)}$, t=1,2,…,n. $\theta_{n,m}$ satisfies the property that $\theta_{n,m}\left(t\right)=\theta_{n,m}\left(t^{\prime }\right)>0$ implies $t=t^{\prime }$.

Then, according to the sensor observation model in Equation (9), the likelihood $\gamma_{k}\left(\left.Z_{k}^{m}\right|X_{k}^{n}\right)$ with Poisson clutter and missed detection can be denoted as [2]:

(13) $$\gamma_{k}\left(\left.Z_{k}^{m}\right|X_{k}^{n}\right)=e^{-\lambda_{k}}\kappa_{k}^{Z_{k}^{m}}\sum_{\theta_{n,m}}\prod_{t=1}^{n}G_{k}\left(\left.z_{k}^{\left(\theta_{n,m}\left(t\right)\right)}\right|x_{k}^{\left(t\right)}\right)$$

where the summation is taken over all association hypotheses $\theta_{n,m}$, and $G_{k}\left(\left.z_{k}^{\left(\theta_{n,m}\left(t\right)\right)}\right|x_{k}^{\left(t\right)}\right)$ is defined as:

(14) $$G_{k}\left(\left.z_{k}^{\left(\theta_{n,m}\left(t\right)\right)}\right|x_{k}^{\left(t\right)}\right)=\left\{\begin{array}{c} \begin{array}{cc} \frac{p_{D,k}\left(x_{k}^{\left(t\right)}\right)g_{k}\left(\left.z_{k}^{\left(\theta_{n,m}\left(t\right)\right)}\right|x_{k}^{\left(t\right)}\right)}{\kappa_{k}\left(z_{k}^{\left(\theta_{n,m}\left(t\right)\right)}\right)}, & \theta_{n,m}\left(t\right)>0 \end{array}\\ \begin{array}{cc} 1-p_{D,k}\left(x_{k}^{\left(t\right)}\right), & \theta_{n,m}\left(t\right)=0 \end{array} \end{array}\right.$$

while the notation $\kappa^{Z}$ denotes:

(15) $$\kappa^{Z}=\left\{\begin{array}{c} \begin{array}{cc} \prod_{z\in Z}\kappa (z), & Z\neq \emptyset \end{array}\\ \begin{array}{cc} 1, & Z=\emptyset \end{array} \end{array}\right.$$

For deriving the error bound for multi-target JDE, the following two conditions must be satisfied as in [16]:

1. MAP detection criterion: This is applied to determine the number of targets: given a measurement set $Z_{k}$ at time *k*, the cardinality of the estimated state set ${\hat{X}}_{k}\left(Z_{k}\right)$ is obtained as the maximum of the posterior probabilities $P_{k}\left(\left.|X_{k}|=n\right|Z_{1:k}\right)$:
  (16) $$\hat{n}=\underset{n}{\arg\max}P_{k}\left(\left.|X_{k}|=n\right|Z_{1:k}\right)$$
  The reason for the use of the MAP detection rule will be clearly explained later in Remark 1 after Theorems 1 and 2.
2. Unbiased estimation criterion: This is a necessary condition for applying the CRLB of Equation (7) in the proof of Theorems 1 and 2.

Next, we derive the proposed bound by using multi-Bernoulli or Poisson approximation for multi-target Bayes recursion, which are stated in Assumptions A.1 and A.2, respectively.

- Assumption A.1: At time *k*, the set $\Gamma_{k}$ of spontaneous births is a multi-Bernoulli RFS with the parameter $\Upsilon_{\Gamma ,k}={\left\{\left(r_{\Gamma ,k}^{\left(i\right)},p_{\Gamma ,k}^{\left(i\right)}\right)\right\}}_{i=1}^{M_{\Gamma ,k}}$ (in general, $\Upsilon_{\Gamma ,k}$ is known *a priori*). Then, the predicted and posterior multi-target densities $f_{k|k-1}\left(\left.X_{k}\right|Z_{1:k-1}\right)$ and $f_{k}\left(\left.X_{k}\right|Z_{1:k}\right)$ are approximated as the multi-Bernoulli densities with parameters $\Upsilon_{k|k-1}={\left\{\left(r_{k|k-1}^{\left(i\right)},p_{k|k-1}^{\left(i\right)}\right)\right\}}_{i=1}^{M_{k|k-1}}$ and $\Upsilon_{k}\;=\;{\left\{\left(r_{k}^{\left(i\right)},p_{k}^{\left(i\right)}\right)\right\}}_{i=1}^{M_{k}}$, respectively. Specifically, the parameter of a multi-Bernoulli RFS that approximates the multi-target RFS is propagated under this assumption. The recursions for $\Upsilon_{k|k-1}$ and $\Upsilon_{k}$ have been presented in [6].
- Assumption A.2: At time *k*, the set $\Gamma_{k}$ of spontaneous births is a Poisson RFS with the intensity $\upsilon_{\Gamma ,k}\left(x_{k}\right)$ (in general, $\upsilon_{\Gamma ,k}\left(x_{k}\right)$ is known *a priori*). Then, the predicted and posterior multi-target densities $f_{k|k-1}\left(\left.X_{k}\right|Z_{1:k-1}\right)$ and $f_{k}\left(\left.X_{k}\right|Z_{1:k}\right)$ are approximated as the Poisson densities with intensities $\upsilon_{k|k-1}\left(x_{k}\right)$ and $\upsilon_{k}\left(x_{k}\right)$, respectively. Specifically, the intensity of a Poisson RFS that approximates the multi-target RFS is propagated under this assumption. The recursions for $\upsilon_{k|k-1}\left(x_{k}\right)$ and $\upsilon_{k}\left(x_{k}\right)$ have been presented in [4].

**Theorem 1.**  *Suppose that Assumption A.1 holds; at time k, given the predicted multi-target multi-Bernoulli parameter $\Upsilon_{k|k-1}={\left\{\left(r_{k|k-1}^{\left(i\right)},p_{k|k-1}^{\left(i\right)}\right)\right\}}_{i=1}^{M_{k|k-1}}$, the error for joint MAP detection and unbiased estimation of multiple targets with the state model in Equation (8) and the sensor observation model in Equation (9) is bounded by:*

(17) $$\begin{array}{c} \sigma_{k}^{2}\geq \sum_{m=0}^{\infty }\sum_{n=0}^{N}\sum_{\hat{n}=0,n+\hat{n}>0}^{N}\frac{\Omega_{k}^{n,m}}{m!\cdot n!\cdot \max\left(n,\hat{n}\right)}\cdot \\ \;\;\;\;\;\;\;\;\left(\sum_{t=1}^{\min\left(n,\hat{n}\right)}\min\left(c^{2}\omega_{k}^{\hat{n},n,m},\sum_{l=1}^{L}{\left[{\left(J_{k}^{\left(t\right),\hat{n},n,m}\right)}^{-1}\right]}_{l,l}\right)+c^{2}\omega_{k}^{\hat{n},n,m}\left|n-\hat{n}\right|\right) \end{array}$$

*where:*

1. *c is the cut-off of the second-order OSPA distance in Equation (4), L is the dimension of state $x_{k}$ and N is the maximum number of the targets observed by the sensor over the surveillance region;*
2. *$\Omega_{k}^{n,m}$ is a normalization factor of the density $f_{k}\left(\left.X_{k}^{n},Z_{k}^{m}\right|Z_{1:k-1}\right)$; it actually denotes the probability of $|X_{k}|=n$ and $|Z_{k}|=m$ given $Z_{1:k-1}$,*
  (18) $$\Omega_{k}^{n,m}=\int_{Z_{k}^{m}}\int_{X_{k}^{n}}f_{k}\left(\left.X_{k}^{n},Z_{k}^{m}\right|Z_{1:k-1}\right)dx_{k}^{\left(1\right)}\cdots dx_{k}^{\left(n\right)}dz_{k}^{\left(1\right)}\cdots dz_{k}^{\left(m\right)}$$
3. *$\omega_{k}^{\hat{n},n,m}$ is the integration of the density $f_{k}\left(\left.X_{k}^{n},Z_{k}^{m}\right|Z_{1:k-1}\right)$ over the region $X_{k}^{n}\times Z_{k}^{\hat{n},m}$,*
  (19) $$\omega_{k}^{\hat{n},n,m}=\int_{Z_{k}^{\hat{n},m}}\int_{X_{k}^{n}}f_{k}\left(\left.X_{k}^{n},Z_{k}^{m}\right|Z_{1:k-1}\right)dx_{k}^{\left(1\right)}\cdots dx_{k}^{\left(n\right)}dz_{k}^{\left(1\right)}\cdots dz_{k}^{\left(m\right)}$$
  *Note that the integration region $Z_{k}^{\hat{n},m}$ in $\omega_{k}^{\hat{n},n,m}$ is the subspace in $Z_{k}^{m}$, where the MAP detector assigns the estimated target number to be $\hat{n}$ ($\hat{n}=0,1,\ldots ,N$). $Z_{k}^{0,m},Z_{k}^{1,m},\ldots ,Z_{k}^{N,m}$ are mutually disjoint and cover $Z_{k}^{m}$. Therefore, $\omega_{k}^{\hat{n},n,m}$ actually denotes the probability of $|X_{k}|=n$ and $|Z_{k}|=m$ given $|{\hat{X}}_{k}|=\hat{n}$ and $Z_{1:k-1}$.*
4. *$J_{k}^{\left(t\right),\hat{n},n,m}$ is the Fisher information matrix of the t-th target given $|Z_{k}|=m$, $|X_{k}|=n$, $|{\hat{X}}_{k}|=\hat{n}$ and $Z_{1:k-1}$. $J_{k}^{\left(t\right),\hat{n},n,m}$, $\omega_{k}^{\hat{n},n,m}$ and $\Omega_{k}^{n,m}$ in Equation (17) are given by (assuming $J_{k}^{\left(t\right),\hat{n},n,m}=\infty$ for $Z_{k}^{\hat{n},m}=\emptyset$, $\hat{n}=0,1,\ldots ,N$):*
  (20) $$\begin{array}{c} {\left[J_{k}^{\left(t\right),\hat{n},n,m}\right]}_{i,j}=\frac{-1}{{\left(\omega_{k}^{\hat{n},n,m}\right)}^{2}}\int_{Z_{k}^{\hat{n},m}}\int_{X_{k}}f_{k}\left(\left.x_{k}^{\left(t\right)},Z_{k}^{m}\right|Z_{1:k-1},\left|X_{k}\right|=n\right)\cdot \\ \;\;\;\;\;\;\;\;\;\;\;\;\;\;\;\;\;\;\;\;\;\;\;\;\frac{\partial^{2}\log f_{k}\left(\left.x_{k}^{\left(t\right)},Z_{k}^{m}\right|Z_{1:k-1},\left|X_{k}\right|=n\right)}{\partial x_{k,i}^{\left(t\right)}\partial x_{k,j}^{\left(t\right)}}dx_{k}^{\left(t\right)}dz_{k}^{\left(1\right)}\cdots dz_{k}^{\left(m\right)} \end{array}$$
  (21) $$\omega_{k}^{\hat{n},n,m}=\frac{\pi_{k|k-1}\left(\emptyset \right)e^{-\lambda_{k}}}{\Omega_{k}^{n,m}}\sum_{\theta_{n,m}}\sum_{1\leq j_{1}\neq \cdot \cdot \cdot \neq j_{n}\leq M_{k|k-1}}\int_{Z_{k}^{\hat{n},m}}\kappa_{k}^{Z_{k}^{m}}D_{k}^{j_{1},\ldots ,j_{n}}\left(z_{k}^{\left(\theta_{n,m}\left(1\right)\right)},\ldots ,z_{k}^{\left(\theta_{n,m}\left(n\right)\right)}\right)dz_{k}^{\left(1\right)}\cdots dz_{k}^{\left(m\right)}$$
  (22) $$\Omega_{k}^{n,m}=\pi_{k|k-1}\left(\emptyset \right)e^{-\lambda_{k}}\lambda_{k}^{m}\sum_{\theta_{n,m}}\sum_{1\leq j_{1}\neq \cdot \cdot \cdot \neq j_{n}\leq M_{k|k-1}}\prod_{t=1}^{n}\frac{r_{k|k-1}^{\left(j_{t}\right)}}{1-r_{k|k-1}^{\left(j_{t}\right)}}K_{k|k-1}^{\left(j_{t}\right)}$$
  (23) $$D_{k}^{j_{1},\ldots ,j_{n}}\left(z_{k}^{\left(\theta_{n,m}\left(1\right)\right)},\ldots ,z_{k}^{\left(\theta_{n,m}\left(n\right)\right)}\right)=\prod_{t=1}^{n}\frac{r_{k|k-1}^{\left(j_{t}\right)}}{1-r_{k|k-1}^{\left(j_{t}\right)}}H_{k}^{j_{t}}\left(z_{k}^{\left(\theta_{n,m}\left(t\right)\right)}\right)$$
  (24) $$H_{k}^{j_{t}}\left(z_{k}^{\left(\theta_{n,m}\left(t\right)\right)}\right)=\int_{X_{k}}p_{k|k-1}^{\left(j_{t}\right)}\left(x_{k}^{\left(t\right)}\right)G_{k}\left(\left.z_{k}^{\left(\theta_{n,m}\left(t\right)\right)}\right|x_{k}^{\left(t\right)}\right)dx_{k}^{\left(t\right)}$$
  (25) Kk|k−1(jt)=∫XkpD,kxk(t)pk|k−1(jt)xk(t)dxk(t)∫XkpD,kxk(t)pk|k−1(jt)xk(t)dxk(t)λkλk,θn,m(t)>0∫Xk1−pD,kxk(t)pk|k−1(jt)xk(t)dxk(t),θn,m(t)=0
  (26) $$\pi_{k|k-1}\left(\emptyset \right)=\prod_{t=1}^{M_{k|k-1}}\left(1-r_{k|k-1}^{\left(t\right)}\right)$$
  *where $G_{k}\left(\left.z_{k}^{\left(\theta_{n,m}\left(t\right)\right)}\right|x_{k}^{\left(t\right)}\right)$ is given by Equation (14), $f_{k}\left(\left.x_{k}^{\left(t\right)},Z_{k}^{m}\right|Z_{1:k-1},\left|X_{k}\right|=n\right)$ is the density of $\left(x_{k}^{\left(t\right)},Z_{k}^{m}\right)$ conditioned on $Z_{1:k-1}$ and $\left|X_{k}\right|=n$. $f_{k}\left(\left.x_{k}^{\left(t\right)},Z_{k}^{m}\right|Z_{1:k-1},\left|X_{k}\right|=n\right)$ in Equation (20), as well as the integration region $Z_{k}^{\hat{n},m}$ in Equations (20) and (21) are given by:*
  (27) $$\begin{array}{c} f_{k}\left(\left.x_{k}^{\left(t\right)},Z_{k}^{m}\right|Z_{1:k-1},\left|X_{k}\right|=n\right)=\\ \frac{\pi_{k|k-1}\left(\emptyset \right)e^{-\lambda_{k}}\kappa_{k}^{Z_{k}^{m}}}{\Omega_{k}^{n,m}}\sum_{\theta_{n,m}}\sum_{1\leq j_{1}\neq \cdot \cdot \cdot \neq j_{n}\leq M_{k|k-1}}\frac{D_{k}^{j_{1},\ldots ,j_{n}}\left(z_{k}^{\left(\theta_{n,m}\left(1\right)\right)},\ldots ,z_{k}^{\left(\theta_{n,m}\left(n\right)\right)}\right)}{H_{k}^{j_{t}}\left(x_{k}^{\left(\theta_{n,m}\left(t\right)\right)}\right)}p_{k|k-1}^{\left(j_{t}\right)}\left(x_{k}^{\left(t\right)}\right)G_{k}\left(\left.z_{k}^{\left(\theta_{n,m}\left(t\right)\right)}\right|x_{k}^{\left(t\right)}\right) \end{array}$$
  (28) $$Z_{k}^{\hat{n},m}=\left\{\begin{array}{cc} Z_{k}^{m}\in Z_{k}^{m}: & \underset{n}{\arg\max}\left\{\xi_{k}^{n}\left(\left.Z_{k}^{m}\right|Z_{1:k-1}\right)\right\}=\hat{n} \end{array}\right\}$$
  (29) $$\begin{array}{c} \xi_{k}^{n}\left(\left.Z_{k}^{m}\right|Z_{1:k-1}\right)=\left(\sum_{1\leq j_{1}\neq \cdot \cdot \cdot \neq j_{n}\leq M_{k|k-1}}\prod_{t=1}^{n}\frac{r_{k|k-1}^{\left(j_{t}\right)}}{1-r_{k|k-1}^{\left(j_{t}\right)}}\right)\cdot \\ \;\;\;\;\;\;\;\;\;\;\;\;\;\;\;\;\;\;\;\;\;\;\;\;\;\;\;\;\;\;\;\sum_{\theta_{n,m}}\sum_{1\leq j_{1}\neq \cdot \cdot \cdot \neq j_{n}\leq M_{k|k-1}}D_{k}^{j_{1},...,j_{n}}\left(z_{k}^{\left(\theta_{n,m}\left(1\right)\right)},...,z_{k}^{\left(\theta_{n,m}\left(n\right)\right)}\right) \end{array}$$
  *where $\xi_{k}^{n}\left(\left.Z_{k}^{m}\right|Z_{1:k-1}\right)$ denotes a function of $Z_{k}^{m}$ and n given $Z_{1:k-1}$.*

**Theorem 2.**  *Suppose that Assumption A.2 holds; at time k, given the predicted multi-target Poisson intensity $\upsilon_{k|k-1}\left(x_{k}\right)$, the error bound for joint MAP detection and unbiased estimation of multiple targets with the state model in Equation (8) and the sensor observation model in Equation (9) takes the same form as in Theorem 1, except that $\omega_{k}^{\hat{n},n,m}$, $\Omega_{k}^{n,m}$, $f_{k}\left(\left.x_{k}^{\left(t\right)},Z_{k}^{m}\right|Z_{1:k-1},\left|X_{k}\right|=n\right)$ and $\xi_{k}^{n}\left(\left.Z_{k}^{m}\right|Z_{1:k-1}\right)$ are changed to:*

(30) $$\omega_{k}^{\hat{n},n,m}=\frac{e^{-\eta_{k|k-1}-\lambda_{k}}}{\Omega_{k}^{n,m}}\sum_{\theta_{n,m}}\int_{Z_{k}^{\hat{n},m}}\kappa_{k}^{Z_{k}^{m}}D_{k}\left(z_{k}^{\left(\theta_{n,m}\left(1\right)\right)},\ldots ,z_{k}^{\left(\theta_{n,m}\left(n\right)\right)}\right)dz_{k}^{\left(1\right)}\cdots dz_{k}^{\left(m\right)}$$

(31) $$\Omega_{k}^{n,m}=e^{-\eta_{k|k-1}-\lambda_{k}}\lambda_{k}^{m}\sum_{\theta_{n,m}}\prod_{t=1}^{n}K_{k|k-1}^{\left(t\right)}$$

(32) $$\begin{array}{c} f_{k}\left(\left.x_{k}^{\left(t\right)},Z_{k}^{m}\right|Z_{1:k-1},\left|X_{k}\right|=n\right)=\frac{e^{-\eta_{k|k-1}-\lambda_{k}}\kappa_{k}^{Z_{k}^{m}}}{\Omega_{k}^{n,m}}\sum_{\theta_{n,m}}\frac{D_{k}\left(z_{k}^{\left(\theta_{n,m}\left(1\right)\right)},\ldots ,z_{k}^{\left(\theta_{n,m}\left(n\right)\right)}\right)}{H_{k}\left(z_{k}^{\left(\theta_{n,m}\left(t\right)\right)}\right)}\cdot \\ \;\;\;\;\;\;\;\;\;\;\;\;\;\;\;\;\;\;\;\;\;\;\;\;\;\;\;\;\;\;\;\;\;\;\;\;\;\;\;\;\;\;\;\;\;\;\;\;\;\;\;\;\;\upsilon_{k|k-1}\left(x_{k}^{\left(t\right)}\right)G_{k}\left(\left.z_{k}^{\left(\theta_{n,m}\left(t\right)\right)}\right|x_{k}^{\left(t\right)}\right) \end{array}$$

(33) $$\xi_{k}^{n}\left(\left.Z_{k}^{m}\right|Z_{1:k-1}\right)={\left(\eta_{k|k-1}\right)}^{n}\sum_{\theta_{n,m}}D_{k}\left(z_{k}^{\left(\theta_{n,m}\left(1\right)\right)},\ldots ,z_{k}^{\left(\theta_{n,m}\left(n\right)\right)}\right)$$

*where:*

(34) $$D_{k}\left(z_{k}^{\left(\theta_{n,m}\left(1\right)\right)},\ldots ,z_{k}^{\left(\theta_{n,m}\left(n\right)\right)}\right)=\prod_{t=1}^{n}H_{k}\left(z_{k}^{\left(\theta_{n,m}\left(t\right)\right)}\right)$$

(35) $$H_{k}\left(z_{k}^{\left(\theta_{n,m}\left(t\right)\right)}\right)=\int_{X_{k}}\upsilon_{k|k-1}\left(x_{k}^{\left(t\right)}\right)G_{k}\left(\left.z_{k}^{\left(\theta_{n,m}\left(t\right)\right)}\right|x_{k}^{\left(t\right)}\right)dx_{k}^{\left(t\right)}$$

(36) Kk|k−1(t)=∫XkpD,kxk(t)υk|k−1xk(t)dxk(t)∫XkpD,kxk(t)υk|k−1xk(t)dxk(t)λkλk,θn,m(t)>0∫Xk1−pD,kxk(t)υk|k−1xk(t)dxk(t),θn,m(t)=0

(37) $$\eta_{k|k-1}=\int \upsilon_{k|k-1}\left(x_{k}\right)dx_{k}$$

The proofs of Theorems 1 and 2 can be found in Appendix A and Appendix B. In the following, we refer to the bound in Theorem 1 or 2 as the multi-Bernoulli approximated bound (MBA-B) or the Poisson approximated bound (PA-B), respectively.

- Remark 1: It is well-known that the lower bound is independent of the specific estimation methods. However, it is necessary for the use of the MAP detection rule in deriving the bounds in Theorems 1 and 2. The reasons are as follows.
  First, we have known that the error metric $e_{k}\left(X_{k},{\hat{X}}_{k}\left(Z_{1:k}\right)\right)$ in Equation (11) is the second-order OSPA distance in Equation (4). Obviously, the estimated target number has to be considered in the OSPA distance. At time *k*, the estimated target number depends on the measurement set $Z_{k}$ received by the sensor. We assume that if $Z_{k}\in Z_{k}^{\hat{n}}$, which is a subspace of the measurement space $Z_{k}$, then the estimated target number by the detector is $\hat{n}$ ($\hat{n}=0,1,\ldots ,N$). Therefore, to compute the MSE $\sigma_{k}^{2}$ in Equation (11), we have to partition the measurement space $Z_{k}$ into the regions of $Z_{k}^{0},Z_{k}^{1},\ldots ,Z_{k}^{N}$, which correspond to all possible estimated target numbers $\hat{n}=0,\hat{n}=1,\ldots ,\hat{n}=N$, respectively. In addition, $Z_{k}^{0},Z_{k}^{1},\ldots ,Z_{k}^{N}$ are mutually disjoint and cover $Z_{k}$.
  In the proof of Theorems 1 and 2, to obtain the bound on $\sigma_{k}^{2}$ in Equation (A13) (Equation (A13) is the extended form of the MSE $\sigma_{k}^{2}$ in Equation (11)), we need to find the best integration regions $Z_{k}^{0,m},Z_{k}^{1,m},\ldots ,Z_{k}^{N,m}$ in Equation (A14) that minimizes Equation (A14). Nevertheless, it is very difficult to define $Z_{k}^{0,m},Z_{k}^{1,m},\ldots ,Z_{k}^{N,m}$ for the detector without using the MAP criterion because the minimization of Equation (A14) depends on the estimator ${\hat{X}}_{k}\left(\cdot \right)$. This reflects the extreme complexity in defining $Z_{k}^{0,m},Z_{k}^{1,m},\ldots ,Z_{k}^{N,m}$ for the detector that minimizes the $\sigma_{k}^{2}$ in Equation (11) and its intricate interconnection with the estimator that may jointly achieve a lower $\sigma_{k}^{2}$ using the MAP detector. A detailed analysis is presented in [16] to illustrate the complicated dependency of the detector and estimator for minimizing the MSE $\sigma_{k}^{2}$. As a result, without the MAP detector restriction, it is nearly impossible to characterize the joint detector-estimator that minimizes the MSE $\sigma_{k}^{2}$ in Equation (11) due to their extremely complex interrelationship in determining the number of targets and estimating the states of existing targets.
  In summary, with the MAP detection constraint, the estimated target number at time *k* can be determined just by the detector (that is, independent of the estimator). However, this may make the minimum MSE defined by Equation (11) unachievable. Therefore, imposing the MAP constraint can be regarded as an approximated method to obtain the proposed JDE bounds. In our future work, we will study the JDE error bound without the MAP detection constraint.
- Remark 2: In general, the integration region $Z_{k}^{\hat{n},m}$ for calculating $J_{k}^{\left(t\right),\hat{n},n,m}$ and $\omega_{k}^{\hat{n},n,m}$ at time *k* is different from the previous integration region $Z_{k-1}^{{\hat{n}}^{\prime },m^{\prime }}$ for calculating $J_{k-1}^{\left(t^{\prime }\right),{\hat{n}}^{\prime },n^{\prime },m^{\prime }}$ and $\omega_{k-1}^{{\hat{n}}^{\prime },n^{\prime },m^{\prime }}$ at time k−1, where the superscripts $t,\hat{n},n,m$ and $t^{\prime },{\hat{n}}^{\prime },n^{\prime },m^{\prime }$ denote the target indices, estimated target numbers, true target numbers and sensor measurement numbers at time *k* and time k−1, respectively. As a result, $J_{k}^{\left(t\right),\hat{n},n,m}$ cannot be derived directly from $J_{k-1}^{\left(t^{\prime }\right),{\hat{n}}^{\prime },n^{\prime },m^{\prime }}$ by using a closed-form recursion like the posterior CRLB (PCRLB) in [11]. The recursion of $J_{k}^{\left(t\right),\hat{n},n,m}$ depends on the propagation of parameter $\Upsilon_{k|k-1}$ or intensity $\upsilon_{k|k-1}\left(x_{k}\right)$ of multi-Bernoulli or Poisson RFS that approximates the predicted multi-target RFS.
- Remark 3: In the special case of no clutter or missed detection, we have $K_{k}=\emptyset$ and $p_{D,k}\left(\cdot \right)=\;1$ for the sensor observation model in Equation (9). The numbers of estimated targets, true targets and measurements are obviously equal in this case, $|{\hat{X}}_{k}\left|=\right|X_{k}\left|=\right|Z_{k}|$. As a result, multi-target JDE reduces to multi-target state estimation only (that is, target detection no longer exists here, and so, the restriction of MAP detection can be omitted) using the sensor measurement. Moreover, given multi-target state set $X_{k}^{n}$, the total likelihood reduces to:
  (38) $$\gamma_{k}\left(\left.Z_{k}^{n}\right|X_{k}^{n}\right)=\sum_{\tau \in \Pi_{n}}\prod_{t=1}^{n}g_{k}\left(\left.z_{k}^{\left(\tau (t)\right)}\right|x_{k}^{\left(t\right)}\right)$$
  and the second-order OSPA distance reduces to:
  (39) $$d_{k}^{2}\left(X_{k}^{n},{\hat{X}}_{k}^{n}\right)=\left\{\begin{array}{c} \begin{array}{cc} 0, & n=0 \end{array}\\ \begin{array}{cc} \frac{1}{n}\min_{\tau \in \Pi_{n}}\sum_{t=1}^{n}{∥x_{k}^{\left(t\right)}-{\hat{x}}_{k}^{\left(\tau (t)\right)}∥}_{2}^{2}, & n>0 \end{array} \end{array}\right.$$
  because there is no need to consider the cut-off *c* for cardinality mismatches here. Only for the special case, a theoretically rigorous (that is, without multi-Bernoulli or Poisson approximation to multi-target Bayes recursion) single-sensor multi-target error bound can be derived in [18] using a PCRLB-like recursion.

## 4. Numerical Examples

A maximum of 10 targets appears on a two-dimensional region S=[−50,50]×[−50,50] (in m) with various births and deaths. The targets are observed by a single sensor with clutter and missed detection throughout a surveillance period of T=25 time steps. The sensor sampling interval is Δt=1s. At time *k*, the state of a target is $x_{k}={\left[x_{k},y_{k},{\dot{x}}_{k},{\dot{y}}_{k},{\ddot{x}}_{k},{\ddot{y}}_{k}\right]}^{T}$, where ${\left[x_{k},y_{k}\right]}^{T}$, ${\left[{\dot{x}}_{k},{\dot{y}}_{k}\right]}^{T}$ and ${\left[{\ddot{x}}_{k},{\ddot{y}}_{k}\right]}^{T}$ denote the position, velocity and acceleration vectors along the *x* axis and *y* axis, respectively. The state transition density $f_{k|k-1}\left(\left.x_{k}\right|x_{k-1}\right)$ is assumed to be:

(40) $$f_{k|k-1}\left(\left.x_{k}\right|x_{k-1}\right)=N\left(x_{k};F_{k}x_{k-1},Q_{k}\right)$$

where $N\left(\cdot ;m,Q\right)$ denotes the density of a Gaussian distribution with mean *m* and covariance matrix *Q* and $F_{k}$ and $Q_{k}$ are the state evolution matrix and process noise covariance matrix at time *k*, respectively. Assuming that the kinematics of each target is governed by the constant acceleration (CA) model [22], we have:

(41) $$\begin{array}{cc} F_{k}=\left[\begin{array}{ccc} 1 & \Delta t & \frac{\Delta t^{2}}{2}\\ 0 & 1 & \Delta t\\ 0 & 0 & 1 \end{array}\right]⊗I_{2}, & Q_{k}=q_{CA}^{2}\left[\begin{array}{ccc} \frac{\Delta t^{4}}{4} & \frac{\Delta t^{3}}{2} & \frac{\Delta t^{2}}{2}\\ \frac{\Delta t^{3}}{2} & \frac{\Delta t^{2}}{2} & \Delta t\\ \frac{\Delta t^{2}}{2} & \Delta t & 1 \end{array}\right]⊗I_{2} \end{array}$$

where ⊗ denotes the Kronecker product, $I_{n}$ is the identity matrix of dimension *n* and $q_{CA}\;=\;0.01\;m/s^{2}$ is the standard deviation of process noise, *i.e*., acceleration. Target births and deaths occur at random instances and states. The probability of target survival is $p_{S,k}\left(\cdot \right)=0.9$. The state of a target birth satisfies one of the distributions $p_{\Gamma }^{\left(i\right)}\left(x_{k}\right)=N\left(x_{k};x_{\Gamma }^{\left(i\right)},Q_{\Gamma }\right)$ (i=1,…,4), $x_{\Gamma }^{\left(1\right)}={\left[20,20,-2,-2,0.1,0.1\right]}^{T}$, $x_{\Gamma }^{\left(2\right)}={\left[20,-20,-2,3,0.1,-0.1\right]}^{T}$, $x_{\Gamma }^{\left(3\right)}={\left[20,-20,-2,3,0.1,-0.1\right]}^{T}$, $x_{\Gamma }^{\left(4\right)}={\left[20,-20,-2,3,0.1,-0.1\right]}^{T}$, $Q_{\Gamma }=diag\left(25,25,0.25,0.25,0.0025,0.0025\right)$, where diag(·) denotes a diagonal matrix. The sensor measurement model for state $x_{k}$ is:

(42) gkzkxk=Nρkok;xk2+yk2arctanykykxkxk,Rk

where $\rho_{k},o_{k}$ are, respectively, the range and bearing measurements of the target and $R_{k}=diag\left(\varsigma_{\rho }^{2},\varsigma_{o}^{2}\right)$ is the sensor measurement noise covariance matrix. In this example, we assume that $\varsigma_{\rho }=2.5\;m$, $\varsigma_{o}=0.1\;\text{rad}$. The detection probability of the sensor is $p_{D,k}\left(\cdot \right)=p_{D}$. The average clutter number and the density of the clutter are $\lambda_{k}=\lambda$ and $f_{c,k}\left(z_{k}\right)=U\left(z_{k};S\right)$, where $U\left(\cdot ;S\right)=1/10^{4}\;$ denotes the density of a uniform distribution over the region S.

For Assumption A.1, the parameter for the multi-Bernoulli set $\Gamma_{k}$ of spontaneous births is $\Upsilon_{\Gamma }\;=\;{\left\{\left(0.1,p_{\Gamma }^{\left(i\right)}\right)\right\}}_{i=1}^{4}$. For Assumption A.2, the intensity for the Poisson set $\Gamma_{k}$ of spontaneous births is $\upsilon_{\Gamma }\left(x_{k}\right)=\sum_{i=1}^{4}0.1p_{\Gamma }^{\left(i\right)}\left(x_{k}\right).$

Then, the proposed bound (MBA-B or PA-B) in this example can be easily obtained by substituting these parameters into Theorem 1 or 2. The second partial derivative $\partial^{2}\log f_{k}\left(\left.x_{k}^{\left(t\right)},Z_{k}^{m}\right|Z_{1:k-1},\left|X_{k}\right|=n\right)/\partial x_{k,i}^{\left(t\right)}\partial x_{k,j}^{\left(t\right)}\;$ involved in Equation (20) can conveniently be obtained by using the software Mathematica 8.0.1. The Monte Carlo (MC) method [23] is used to numerically calculate ${\left[J_{k}^{\left(t\right),\hat{n},n,m}\right]}_{i,j}$ and $\omega_{k}^{\hat{n},n,m}$ because the involved integrals in them have no closed-forms.

**Figure 1:**
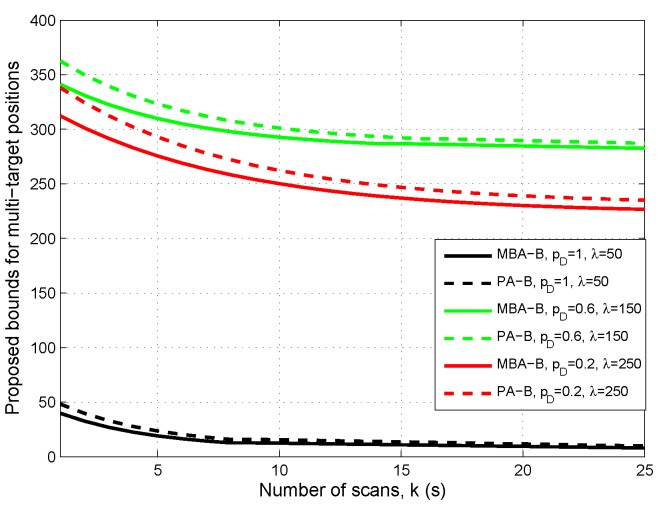
Proposed bounds for multi-target positions *versus* scan in the cases: $p_{D}=1,\;\lambda =50$ (black lines); $p_{D}=0.6,\;\lambda =150$ (green lines); $p_{D}=0.2,\;\lambda =250$ (red lines).

First, let us see how the sensor measurement uncertainty would affect the proposed bound. It is clear that the measurement uncertainty of a sensor is mainly determined by its detection probability and clutter. Therefore, in Figure 1, the proposed two bounds of multi-target position vectors are shown *versus* scan for three groups of detection probability and clutter intensity: (1) $p_{D}=1,\;\lambda =50$, (2) $p_{D}=0.6,\;\lambda =150$ and (3) $p_{D}=0.2,\;\lambda =250$, respectively, where the cut-off of OSPA distance is $c^{2}=400$.

From Figure 1, it can be seen that both bounds are asymptotically convergent for various $p_{D}$ and *λ*. As the number of sensor measurement scans increases, they will get closer. The bounds for the case $p_{D}=1,\;\lambda =50$ are the smallest in the three cases. However, it is somewhat surprising that the bounds for the case $p_{D}=0.2,\;\lambda =250$ are lower than the bounds for the case $p_{D}=0.6,\;\lambda =150$. Moreover, the bigger *λ* becomes for $p_{D}$, or the lower $p_{D}$ becomes for *λ*, the longer the convergence time of the bounds seems to be. Figure 1 indicates that clutter density and detection probability of the sensor do have a significant impact on the proposed bound.

**Figure 2:**
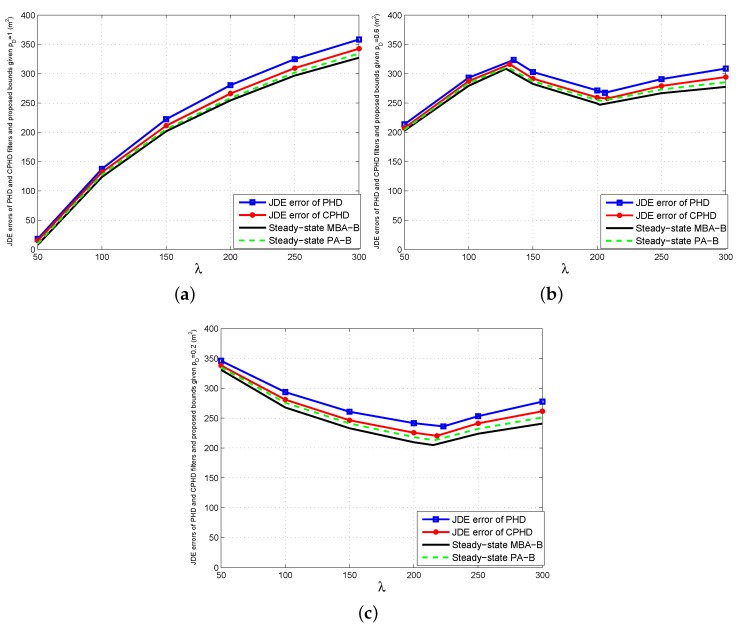
Comparisons of joint detection and estimation (JDE) errors of single-sensor probability hypothesis density (PHD) and cardinalized PHD (CPHD) filters with steady-state bounds for multi-target positions. (**a**) $p_{D}=1$; (**b**) $p_{D}=0.6$; (**c**) $p_{D}=0.2$.

To verify the effectiveness of the proposed bounds, we compare the steady-state bounds with the JDE errors of the single-sensor PHD and CPHD filters, which are the average of 200 MC runs of their time-averaged OSPA distances between the true and estimated state sets. The comparison results are presented in Figure 2.

From Figure 2, we can obtain the following observations.

1. The proposed bound does not always increase with *λ* for given $p_{D}$ or decrease with $p_{D}$ for given *λ*. This is because of the two contrary effects generated by the increase of *λ* or $p_{D}$ when $p_{D}<1$ or λ>0: reducing the possibility for missed targets and increasing the possibility for false targets. If the bound is dominated by the former, then it decreases with *λ* or $p_{D}$; otherwise, it increases with *λ* or $p_{D}$. Moreover, PA-B is a little higher than MBA-B when *λ* is relatively large or $p_{D}$ is relatively small. However, they are very close in general. A possible reason for this is that the multi-Bernoulli assumption (Assumption A.1) outperforms the Poisson assumption (Assumption A.2) slightly for approximating the multi-target Bayes recursion under lower signal-noise-ratio (SNR) conditions.
2. Although the JDE errors of the single-sensor PHD and CPHD filters are a little higher than the proposed bound, all of them are always close *versus* *λ* and $p_{D}$. The extra errors of the two filters are generated by the first-order moment approximations for the posterior multi-target density and the clustering processes involved in their particle implementations for state extraction. Figure 2 also shows that the CPHD filter outperforms the PHD filter. The reason for this is that the former can propagate the cardinality distribution and, thus, has more stable target number estimation than the latter.

3. The bigger *λ* becomes for given $p_{D}$, or the lower $p_{D}$ becomes for given *λ*, the bigger the gaps between the errors of the two filters and the proposed bound will be. This is because the aforementioned approximation errors of the two filters increase as *λ* becomes bigger or $p_{D}$ becomes smaller. However, the maximum relative errors of the PHD and CPHD filters, which seem to appear in the case of $p_{D}=0.2$ and λ=300, do not exceed 15% and 8% of MBA-B, as well as 12% and 5% of PA-B in any case, respectively. In fact, the total average relative errors of the two filters are about 7% and 4% of MBA-B, as well as about 6% and 3% of PA-B for various *λ* and $p_{D}$, respectively.
  Finally, the comparison results in Figure 2 show that for various clutter densities and detection probabilities of the sensor, the proposed bounds are able to provide an effective indication of performance limitations for the two single-sensor multi-target JDE algorithms.

## 5. Conclusions

Within the RFS framework, we develop two multi-target JDE error bounds using the measurement of a single sensor with clutter and missed detection. The multi-Bernoulli and Poisson approximation to multi-target Bayes recursion are used in deriving the results of the paper, respectively. The proposed bounds are based on the OSPA distance rather than the Euclidean distance. The simulation results show that the clutter density and detection probability of the sensor significantly affect the bounds and verify the effectiveness of the bounds by indicating the performance limitations of the single-sensor PHD and CPHD filters in various sensor measurement environments.

Our future work will focus on the following four aspects:

1. Extending the results to the case of multiple sensors;
2. Extending the results to the case of the biased estimator by using the ordinary information inequality of Equation (5);
3. Studying the JDE error bounds without the MAP detection constraint;
4. Studying the sensor management strategies based on the results.

## Appendix A

**Proof of Theorem 1.** First, we determine the target number from the sequence of sensor measurements according to the MAP criterion. Given a measurement set $Z_{k}^{m}$ received by the sensor at time *k*, using the Bayes rule on the posterior probability $P_{k}\left(\left.\left|X_{k}\right|=n\right|Z_{1:k-1},Z_{k}^{m}\right)$, we get:

(A1) $$P_{k}\left(\left.\left|X_{k}\right|=n\right|Z_{1:k-1},Z_{k}^{m}\right)=\frac{P_{k}\left(\left.\left|X_{k}\right|=n\right|Z_{1:k-1}\right)P_{k}\left(\left.Z_{k}^{m}\right|\left|X_{k}\right|=n\right)}{P_{k}\left(\left.Z_{k}^{m}\right|Z_{1:k-1}\right)}$$

where $P_{k}\left(\left.Z_{k}^{m}\right|Z_{1:k-1}\right)$ is a normalizing factor, $P_{k}\left(\left.\left|X_{k}\right|=n\right|Z_{1:k-1}\right)$ and $P_{k}\left(\left.Z_{k}^{m}\right|\left|X_{k}\right|=n\right)$ can be obtained by:

(A2) $$P_{k}\left(\left.\left|X_{k}\right|=n\right|Z_{1:k-1}\right)=\int_{X_{k}^{n}}f_{k|k-1}\left(\left.X_{k}^{n}\right|Z_{1:k-1}\right)dx_{k}^{\left(1\right)}\cdots dx_{k}^{\left(n\right)}$$

(A3) $$P_{k}\left(\left.Z_{k}^{m}\right|\left|X_{k}\right|=n\right)=\int_{X_{k}^{n}}f_{k|k-1}\left(\left.X_{k}^{n}\right|Z_{1:k-1}\right)\gamma_{k}\left(Z_{k}^{m}\left|X_{k}^{n}\right.\right)dx_{k}^{\left(1\right)}\cdots dx_{k}^{\left(n\right)}$$

where the likelihood $\gamma_{k}\left(Z_{k}^{m}\left|X_{k}^{n}\right.\right)$ is given by Equation (13) and the predicted multi-target density $f_{k|k-1}\left(\left.X_{k}^{n}\right|Z_{1:k-1}\right)$ is a multi-Bernoulli density with the parameter $\Upsilon_{k|k-1}={\left\{\left(r_{k|k-1}^{\left(t\right)},p_{k|k-1}^{\left(t\right)}\right)\right\}}_{t=1}^{M_{k|k-1}}$ according to Assumption A.1,

(A4) $$f_{k|k-1}\left(\left.X_{k}^{n}\right|Z_{1:k-1}\right)=\pi_{k|k-1}\left(\emptyset \right)\sum_{1\leq j_{1}\neq \cdot \cdot \cdot \neq j_{n}\leq M_{k|k-1}}\prod_{t=1}^{n}\frac{r_{k|k-1}^{\left(j_{t}\right)}}{1-r_{k|k-1}^{\left(j_{t}\right)}}p_{k|k-1}^{\left(j_{t}\right)}\left(x_{k}^{\left(t\right)}\right)$$

where:

(A5) $$\pi_{k|k-1}\left(\emptyset \right)=\prod_{t=1}^{M_{k|k-1}}\left(1-r_{k|k-1}^{\left(t\right)}\right)$$

Substituting Equations (A4) and (13) into Equations (A2) and (A3), respectively, and integrating out $x_{k}^{\left(1\right)},\ldots ,x_{k}^{\left(n\right)}$ in the two equations, we have:

(A6) $$P_{k}\left(\left.\left|X_{k}\right|=n\right|Z_{1:k-1}\right)=\pi_{k|k-1}\left(\emptyset \right)\sum_{1\leq j_{1}\neq \cdot \cdot \cdot \neq j_{n}\leq M_{k|k-1}}\prod_{t=1}^{n}\frac{r_{k|k-1}^{\left(j_{t}\right)}}{1-r_{k|k-1}^{\left(j_{t}\right)}}$$

(A7) $$P_{k}\left(\left.Z_{k}^{m}\right|\left|X_{k}\right|=n\right)=\pi_{k|k-1}\left(\emptyset \right)e^{-\lambda_{k}}\kappa_{k}^{Z_{k}^{m}}\sum_{\theta_{n,m}}\sum_{1\leq j_{1}\neq \cdot \cdot \cdot \neq j_{n}\leq M_{k|k-1}}D_{k}^{j_{1},\ldots ,j_{n}}\left(z_{k}^{\left(\theta_{n,m}\left(1\right)\right)},\ldots ,z_{k}^{\left(\theta_{n,m}\left(n\right)\right)}\right)$$

where:

(A8) $$D_{k}^{j_{1},\ldots ,j_{n}}\left(z_{k}^{\left(\theta_{n,m}\left(1\right)\right)},\ldots ,z_{k}^{\left(\theta_{n,m}\left(n\right)\right)}\right)=\prod_{t=1}^{n}\frac{r_{k|k-1}^{\left(j_{t}\right)}}{1-r_{k|k-1}^{\left(j_{t}\right)}}H_{k}^{j_{t}}\left(z_{k}^{\left(\theta_{n,m}\left(t\right)\right)}\right)$$

(A9) $$H_{k}^{j_{t}}\left(z_{k}^{\left(\theta_{n,m}\left(t\right)\right)}\right)=\int_{X_{k}}p_{k|k-1}^{\left(j_{t}\right)}\left(x_{k}^{\left(t\right)}\right)G_{k}\left(\left.z_{k}^{\left(\theta_{n,m}\left(t\right)\right)}\right|x_{k}^{\left(t\right)}\right)dx_{k}^{\left(t\right)}$$

and $G_{k}\left(\left.z_{k}^{\left(\theta_{n,m}\left(t\right)\right)}\right|x_{k}^{\left(t\right)}\right)$ is given by Equation (14). Substituting Equations (A6) and (A7) into Equation (A1), the posterior probability $P_{k}\left(\left.\left|X_{k}\right|=n\right|Z_{1:k-1},Z_{k}^{m}\right)$ becomes:

(A10) $$P_{k}\left(\left.\left|X_{k}\right|=n\right|Z_{1:k-1},Z_{k}^{m}\right)=\frac{\pi_{k|k-1}^{2}\left(\emptyset \right)e^{-\lambda_{k}}\kappa_{k}^{Z_{k}^{m}}}{P_{k}\left(\left.Z_{k}^{m}\right|Z_{1:k-1}\right)}\xi_{k}^{n}\left(\left.Z_{k}^{m}\right|Z_{1:k-1}\right)$$

where:

$$\begin{array}{c} \xi_{k}^{n}\left(\left.Z_{k}^{m}\right|Z_{1:k-1}\right)=\left(\sum_{1\leq j_{1}\neq \cdot \cdot \cdot \neq j_{n}\leq M_{k|k-1}}\prod_{t=1}^{n}\frac{r_{k|k-1}^{\left(j_{t}\right)}}{1-r_{k|k-1}^{\left(j_{t}\right)}}\right)\\ \cdot \sum_{\theta_{n,m}}\sum_{1\leq j_{1}\neq \cdot \cdot \cdot \neq j_{n}\leq M_{k|k-1}}D_{k}^{j_{1},\ldots ,j_{n}}\left(z_{k}^{\left(\theta_{n,m}\left(1\right)\right)},\ldots ,z_{k}^{\left(\theta_{n,m}\left(n\right)\right)}\right) \end{array}$$

denotes a function of $Z_{k}^{m}$ and *n* given $Z_{1:k-1}$. The MAP detector upon observing $Z_{k}^{m}$ given $Z_{1:k-1}$ will assign $\left|{\hat{X}}_{k}\left(Z_{1:k-1},Z_{k}^{m}\right)\right|=\hat{n}$ if:

(A11) $$\hat{n}=\underset{n}{\arg\max}\left\{P_{k}\left(\left.\left|X_{k}\right|=n\right|Z_{1:k-1},Z_{k}^{m}\right)\right\}=\underset{n}{\arg\max}\left\{\xi_{k}^{n}\left(\left.Z_{k}^{m}\right|Z_{1:k-1}\right)\right\}\Leftrightarrow Z_{k}^{m}\in Z_{k}^{\hat{n},m}$$

where $Z_{k}^{\hat{n},m}$ is the subspace of the *m*-point sensor measurement space $Z_{k}^{m}$. Equation (A12) denotes that, at time *k*, the MAP detector assigns the estimated target number to be $\hat{n}$ ($\hat{n}=0,1,\ldots ,N$, where *N* is the maximum number of targets observed by the sensor over the surveillance region) if receiving the sensor measurement $Z_{k}^{m}\in Z_{k}^{\hat{n},m}$. $Z_{k}^{0,m},Z_{k}^{1,m},\ldots ,Z_{k}^{N,m}$ are mutually disjoint and cover $Z_{k}^{m}$. For the *m*-point measurement space $Z_{k}^{m}$, its partitions $Z_{k}^{0,m},Z_{k}^{1,m},\ldots ,Z_{k}^{N,m}$ correspond to the all possible estimated target numbers $\hat{n}=0,\hat{n}=1,\ldots ,\hat{n}=N$, respectively. According to the set integral definition in Equation (1), the MSE in Equation (11) can be extended as:

(A12) $$\begin{array}{c} \sigma_{k}^{2}=\sum_{m=0}^{\infty }\sum_{n=0}^{N}\frac{1}{m!\cdot n!}\int_{Z_{k}^{m}}\int_{X_{k}^{n}}\gamma_{k}\left(\left.Z_{k}^{m}\right|X_{k}^{n}\right)f_{k|k-1}\left(\left.X_{k}^{n}\right|Z_{1:k-1}\right)\cdot \\ \;\;\;\;\;\;\;\;\;e_{k}^{2}\left(X_{k}^{n},{\hat{X}}_{k}\left(Z_{1:k-1},Z_{k}^{m}\right)\right)dx_{k}^{\left(1\right)}\cdots dx_{k}^{\left(n\right)}dz_{k}^{\left(1\right)}\cdots dz_{k}^{\left(m\right)} \end{array}$$

By partitioning the integration region $Z_{k}^{m}$ in Equation (A13) into sub-regions $Z_{k}^{0,m},Z_{k}^{1,m},\ldots ,Z_{k}^{N,m}$ according to the MAP detector in Equation (A12), we have:

(A13) $$\begin{array}{c} \sigma_{k}^{2}=\sum_{m=0}^{\infty }\sum_{n=0}^{N}\sum_{\hat{n}=0}^{N}\frac{1}{m!\cdot n!}\int_{Z_{k}^{\hat{n},m}}\int_{X_{k}^{n}}\gamma_{k}\left(\left.Z_{k}^{m}\right|X_{k}^{n}\right)f_{k|k-1}\left(\left.X_{k}^{n}\right|Z_{1:k-1}\right)\cdot \\ \;\;\;\;\;\;\;\;\;e_{k}^{2}\left(X_{k}^{n},{\hat{X}}_{k}^{\hat{n}}\left(Z_{1:k-1},Z_{k}^{m}\right)\right)dx_{k}^{\left(1\right)}\cdots dx_{k}^{\left(n\right)}dz_{k}^{\left(1\right)}\cdots dz_{k}^{\left(m\right)} \end{array}$$

Using the Bayes rule on density $f_{k}\left(\left.X_{k}^{n},Z_{k}^{m}\right|Z_{1:k-1}\right)$, we get:

(A14) $$f_{k}\left(X_{k}^{n},Z_{k}^{m}|Z_{1:k-1}\right)=\frac{1}{\Omega_{k}^{n,m}}\gamma_{k}\left(Z_{k}^{m}|X_{k}^{n}\right)f_{k|k-1}\left(X_{k}^{n}|Z_{1:k-1}\right)$$

where $\Omega_{k}^{n,m}$ is a normalization factor, and:

(A15) $$\Omega_{k}^{n,m}=\int_{Z_{k}^{m}}\int_{X_{k}^{n}}f_{k|k-1}\left(\left.X_{k}^{n}\right|Z_{1:k-1}\right)\gamma_{k}\left(\left.Z_{k}^{m}\right|X_{k}^{n}\right)dx_{k}^{\left(1\right)}\cdots dx_{k}^{\left(n\right)}dz_{k}^{\left(1\right)}\cdots dz_{k}^{\left(m\right)}$$

actually denotes the probability of $|X_{k}|=n$ and $|Z_{k}|=m$ given $Z_{1:k-1}$. Substituting Equations (A4) and (13) into Equations (A15) and (A16), respectively, and integrating out $z_{k}^{\left(1\right)},\ldots ,z_{k}^{\left(m\right)}$ in Equation (A16), $f_{k}\left(\left.X_{k}^{n},Z_{k}^{m}\right|Z_{1:k-1}\right)$ becomes:

(A16) $$\begin{array}{c} f_{k}\left(\left.X_{k}^{n},Z_{k}^{m}\right|Z_{1:k-1}\right)=\\ \frac{\pi_{k|k-1}\left(\emptyset \right)e^{-\lambda_{k}}\kappa_{k}^{Z_{k}^{m}}}{\Omega_{k}^{n,m}}\sum_{\theta_{n,m}}\sum_{1\leq j_{1}\neq \cdot \cdot \cdot \neq j_{n}\leq M_{k|k-1}}\prod_{t=1}^{n}\frac{r_{k|k-1}^{\left(j_{t}\right)}}{1-r_{k|k-1}^{\left(j_{t}\right)}}p_{k|k-1}^{\left(j_{t}\right)}\left(x_{k}^{\left(t\right)}\right)G_{k}\left(\left.z_{k}^{\left(\theta_{n,m}\left(t\right)\right)}\right|x_{k}^{\left(t\right)}\right) \end{array}$$

and $\Omega_{k}^{n,m}$ is obtained as:

(A17) $$\Omega_{k}^{n,m}=\pi_{k|k-1}\left(\emptyset \right)e^{-\lambda_{k}}\lambda_{k}^{m}\sum_{\theta_{n,m}}\sum_{1\leq j_{1}\neq \cdot \cdot \cdot \neq j_{n}\leq M_{k|k-1}}\prod_{t=1}^{n}\frac{r_{k|k-1}^{\left(j_{t}\right)}}{1-r_{k|k-1}^{\left(j_{t}\right)}}K_{k|k-1}^{\left(j_{t}\right)}$$

where:

(A18) Kk|k−1(jt)=∫XkpD,kxk(t)pk|k−1(jt)xk(t)dxk(t)∫XkpD,kxk(t)pk|k−1(jt)xk(t)dxk(t)λkλk,θn,m(t)>0∫Xk1−pD,kxk(t)pk|k−1(jt)xk(t)dxk(t),θn,m(t)=0

For Equation (A14), replacing $\gamma_{k}\left(\left.Z_{k}^{m}\right|X_{k}^{n}\right)f_{k|k-1}\left(\left.X_{k}^{n}\right|Z_{1:k-1}\right)$ with $\Omega_{k}^{n,m}f_{k}\left(\left.X_{k}^{n},Z_{k}^{m}\right|Z_{1:k-1}\right)$ according to Equation (A15) and then replacing $e_{k}^{2}\left(X_{k}^{n},{\hat{X}}_{k}^{\hat{n}}\left(Z_{1:k-1},Z_{k}^{m}\right)\right)$ with the second-order OSPA distance defined in Equation (4), we get:

(A19) $$\begin{array}{c} \begin{array}{c} \sigma_{k}^{2}=\sum_{m=0}^{\infty }\sum_{n=0}^{N}\sum_{\hat{n}=0,n+\hat{n}>0}^{N}\frac{\Omega_{k}^{n,m}}{m!\cdot n!\cdot \max(\hat{n},n)}\int_{Z_{k}^{\hat{n},m}}\int_{X_{k}^{n}}f_{k}\left(\left.X_{k}^{n},Z_{k}^{m}\right|Z_{1:k-1}\right)\cdot \\ \left(\min_{\tau \in \Pi_{\max(\hat{n},n)}}\sum_{t=1}^{\min(\hat{n},n)}\min\left(c^{2},{∥x_{k}^{\left(t\right)}-{\hat{x}}_{k}^{\left(\tau (t)\right)}\left(Z_{1:k-1},Z_{k}^{m}\right)∥}_{2}^{2}\right)+c^{2}\left|n-\hat{n}\right|\right)dx_{k}^{\left(1\right)}\cdots dx_{k}^{\left(n\right)}dz_{k}^{\left(1\right)}\cdots dz_{k}^{\left(m\right)} \end{array} \end{array}$$

where *c* is the cut-off of the OSPA distance. Let:

(A20) $$\omega_{k}^{\hat{n},n,m}=\int_{Z_{k}^{\hat{n},m}}\int_{X_{k}^{n}}f_{k}\left(\left.X_{k}^{n},Z_{k}^{m}\right|Z_{1:k-1}\right)dx_{k}^{\left(1\right)}\cdots dx_{k}^{\left(n\right)}dz_{k}^{\left(1\right)}\cdots dz_{k}^{\left(m\right)}$$

denote the integral of the density $f_{k}\left(\left.X_{k}^{n},Z_{k}^{m}\right|Z_{1:k-1}\right)$ over the region $Z_{k}^{\hat{n},m}\times X_{k}^{n}$. $\omega_{k}^{\hat{n},n,m}$ actually denotes the probability of $|X_{k}|=n$ and $|Z_{k}|=m$ given $|{\hat{X}}_{k}|=\hat{n}$ and $Z_{1:k-1}$, where $|{\hat{X}}_{k}|=\hat{n}$ is from the MAP detector of Equation (A12). Replacing $f_{k}\left(\left.X_{k}^{n},Z_{k}^{m}\right|Z_{1:k-1}\right)$ in Equation (A21) with Equation (A17) and integrating out $x_{k}^{\left(1\right)},\ldots ,x_{k}^{\left(n\right)}$, $\omega_{k}^{\hat{n},n,m}$ can be rewritten as:

(A21) $$\begin{array}{c} \begin{array}{c} \omega_{k}^{\hat{n},n,m}=\frac{\pi_{k|k-1}\left(\emptyset \right)e^{-\lambda_{k}}}{\Omega_{k}^{n,m}}\sum_{\theta_{n,m}}\sum_{1\leq j_{1}\neq \cdot \cdot \cdot \neq j_{n}\leq M_{k|k-1}}\int_{Z_{k}^{\hat{n},m}}\kappa_{k}^{Z_{k}^{m}}D_{k}^{j_{1},\ldots ,j_{n}}\left(z_{k}^{\left(\theta_{n,m}\left(1\right)\right)},\ldots ,z_{k}^{\left(\theta_{n,m}\left(n\right)\right)}\right)dz_{k}^{\left(1\right)}\cdots dz_{k}^{\left(m\right)} \end{array} \end{array}$$

Let:

(A22) $$\tau^{*}=\underset{\tau \in \Pi_{\max(\hat{n},n)}}{\arg\min}\left\{\sum_{t=1}^{\min(\hat{n},n)}\min\left(c^{2},{∥x_{k}^{\left(t\right)}-{\hat{x}}_{k}^{\left(\tau (t)\right)}\left(Z_{1:k-1},Z_{k}^{m}\right)∥}_{2}^{2}\right)\right\}$$

denote the permutation in $\Pi_{\max\left(\hat{n},n\right)}$, which minimizes $\sum_{t=1}^{\min\left(\hat{n},n\right)}\min\left(c^{2},{∥x_{k}^{\left(t\right)}-{\hat{x}}_{k}^{\left(\tau (t)\right)}\left(Z_{1:k-1},Z_{k}^{m}\right)∥}_{2}^{2}\right)$. By the use of $\omega_{k}^{\hat{n},n,m}$ defined in Equation (A21) and $\tau^{*}$ defined in Equation (A23), then Equation (A20) can be rewritten as:

(A23) $$\begin{array}{c} \sigma_{k}^{2}=\sum_{m=0}^{\infty }\sum_{n=0}^{N}\sum_{\hat{n}=0,n+\hat{n}>0}^{N}\frac{\Omega_{k}^{n,m}}{m!\cdot n!\cdot \max(\hat{n},n)}\\ \cdot \left(\begin{array}{c} \sum_{t=1}^{\min(\hat{n},n)}\min\left(\begin{array}{c} c^{2}\omega_{k}^{\hat{n},n,m},\int_{Z_{k}^{\hat{n},m}}\int_{X_{k}^{n}}f_{k}\left(\left.X_{k}^{n},Z_{k}^{m}\right|Z_{1:k-1}\right)\cdot \\ {∥x_{k}^{\left(t\right)}-{\hat{x}}_{k}^{\left(\tau^{*}\left(t\right)\right)}\left(Z_{1:k-1},Z_{k}^{m}\right)∥}_{2}^{2}dx_{k}^{\left(1\right)}\cdots dx_{k}^{\left(n\right)}dz_{k}^{\left(1\right)}\cdots dz_{k}^{\left(m\right)} \end{array}\right)\\ +c^{2}\left|n-\hat{n}\right|\omega_{k}^{\hat{n},n,m} \end{array}\right) \end{array}$$

Let $f_{k}\left(\left.x_{k}^{\left(t\right)},Z_{k}^{m}\right|Z_{1:k-1},\left|X_{k}\right|=n\right)$ denote the joint probability density of $\left(x_{k}^{\left(t\right)},Z_{k}^{m}\right)$ conditioned on $Z_{1:k-1}$ and $\left|X_{k}\right|=n$. $f_{k}\left(\left.x_{k}^{\left(t\right)},Z_{k}^{m}\right|Z_{1:k-1},\left|X_{k}\right|=n\right)$ can be obtained by:

(A24) $$f_{k}\left(\left.x_{k}^{\left(t\right)},Z_{k}^{m}\right|Z_{1:k-1},\left|X_{k}\right|=n\right)=\int_{X_{k}^{n-1}}f_{k}\left(\left.X_{k}^{n},Z_{k}^{m}\right|Z_{1:k-1}\right)dx_{k}^{\left(1\right)}\cdots dx_{k}^{\left(t-1\right)}dx_{k}^{\left(t+1\right)}\cdots dx_{k}^{\left(n\right)}$$

Replacing $f_{k}\left(\left.X_{k}^{n},Z_{k}^{m}\right|Z_{1:k-1}\right)$ in Equation (A25) with Equation (A17) and integrating out $x_{k}^{\left(1\right)},\ldots ,x_{k}^{\left(t-1\right)},x_{k}^{\left(t+1\right)},\ldots ,x_{k}^{\left(n\right)}$, $f_{k}\left(\left.x_{k}^{\left(t\right)},Z_{k}^{m}\right|Z_{1:k-1},\left|X_{k}\right|=n\right)$ can be rewritten as:

(A25) $$\begin{array}{c} \begin{array}{c} f_{k}\left(\left.x_{k}^{\left(t\right)},Z_{k}^{m}\right|Z_{1:k-1},\left|X_{k}\right|=n\right)=\\ \frac{\pi_{k|k-1}\left(\emptyset \right)e^{-\lambda_{k}}\kappa_{k}^{Z_{k}^{m}}}{\Omega_{k}^{n,m}}\sum_{\theta_{n,m}}\sum_{1\leq j_{1}\neq \cdot \cdot \cdot \neq j_{n}\leq M_{k|k-1}}\frac{D_{k}^{j_{1},\ldots ,j_{n}}\left(z_{k}^{\left(\theta_{n,m}\left(1\right)\right)},\ldots ,z_{k}^{\left(\theta_{n,m}\left(n\right)\right)}\right)}{H_{k}^{j_{t}}\left(z_{k}^{\left(\theta_{n,m}\left(t\right)\right)}\right)}p_{k|k-1}^{\left(j_{t}\right)}\left(x_{k}^{\left(t\right)}\right)G_{k}\left(\left.z_{k}^{\left(\theta_{n,m}\left(t\right)\right)}\right|x_{k}^{\left(t\right)}\right) \end{array} \end{array}$$

According to the density $f_{k}\left(\left.x_{k}^{\left(t\right)},Z_{k}^{m}\right|Z_{1:k-1},\left|X_{k}\right|=n\right)$ defined in Equation (A25), the integral $\int_{Z_{k}^{\hat{n},m}}\int_{X_{k}^{n}}f_{k}\left(\left.X_{k}^{n},Z_{k}^{m}\right|Z_{1:k-1}\right){∥x_{k}^{\left(t\right)}-{\hat{x}}_{k}^{\left(\tau^{*}\left(t\right)\right)}\left(Z_{1:k-1},Z_{k}^{m}\right)∥}_{2}^{2}dx_{k}^{\left(1\right)}\cdots dx_{k}^{\left(n\right)}dz_{k}^{\left(1\right)}\cdots dz_{k}^{\left(m\right)}$ involved in Equation (A24) can be rewritten as:

(A26) $$\begin{array}{c} \begin{array}{c} \int_{Z_{k}^{\hat{n},m}}\int_{X_{k}^{n}}f_{k}\left(\left.X_{k}^{n},Z_{k}^{m}\right|Z_{1:k-1}\right){∥x_{k}^{\left(t\right)}-{\hat{x}}_{k}^{\left(\tau^{*}\left(t\right)\right)}\left(Z_{1:k-1},Z_{k}^{m}\right)∥}_{2}^{2}dx_{k}^{\left(1\right)}\cdots dx_{k}^{\left(n\right)}dz_{k}^{\left(1\right)}\cdots dz_{k}^{\left(m\right)}\\ =\int_{Z_{k}^{\hat{n},m}}\int_{X_{k}}f_{k}\left(\left.x_{k}^{\left(t\right)},Z_{k}^{m}\right|Z_{1:k-1},\left|X_{k}\right|=n\right)\sum_{l=1}^{L}{\left(x_{k,l}^{\left(t\right)}-{\hat{x}}_{k,l}^{\left(\tau^{*}\left(t\right)\right)}\left(Z_{1:k-1},Z_{k}^{m}\right)\right)}^{2}dx_{k}^{\left(t\right)}dz_{k}^{\left(1\right)}\cdots dz_{k}^{\left(m\right)} \end{array} \end{array}$$

where *L* is the dimension of the state $x_{k}$. Since the estimator has been assumed to be unbiased, we can apply the CRLB of Equation (7) to the density $f_{k}\left(\left.x_{k}^{\left(t\right)},Z_{k}^{m}\right|Z_{1:k-1},\left|X_{k}\right|=n\right)$ in Equation (A27),

(A27) $$\begin{array}{c} \int_{Z_{k}^{\hat{n},m}}\int_{X_{k}}f_{k}\left(\left.x_{k}^{\left(t\right)},Z_{k}^{m}\right|Z_{1:k-1},\left|X_{k}\right|=n\right){\left(x_{k,l}^{\left(t\right)}-{\hat{x}}_{k,l}^{\left(\tau^{*}\left(t\right)\right)}\left(Z_{1:k-1},Z_{k}^{m}\right)\right)}^{2}dx_{k}^{\left(t\right)}dz_{k}^{\left(1\right)}\cdots dz_{k}^{\left(m\right)}\\ \begin{array}{cc} \geq {\left[{\left(J_{k}^{\left(t\right),\hat{n},n,m}\right)}^{-1}\right]}_{l,l}, & l=1,\ldots ,L \end{array} \end{array}$$

where:

(A28) $$\begin{array}{c} {\left[J_{k}^{\left(t\right),\hat{n},n,m}\right]}_{i,j}=\frac{-1}{{\left(\omega_{k}^{\left(t\right),\hat{n},n,m}\right)}^{2}}\int_{Z_{k}^{\hat{n},m}}\int_{X_{k}}f_{k}\left(\left.x_{k}^{\left(t\right)},Z_{k}^{m}\right|Z_{1:k-1},\left|X_{k}\right|=n\right)\cdot \\ \;\;\;\;\;\;\;\;\;\;\;\;\;\;\;\;\;\;\;\;\frac{\partial^{2}\log f_{k}\left(\left.x_{k}^{\left(t\right)},Z_{k}^{m}\right|Z_{1:k-1},\left|X_{k}\right|=n\right)}{\partial x_{k,i}^{\left(t\right)}\partial x_{k,j}^{\left(t\right)}}dx_{k}^{\left(t\right)}dz_{k}^{\left(1\right)}\cdots dz_{k}^{\left(m\right)} \end{array}$$

(A29) $$\omega_{k}^{\left(t\right),\hat{n},n,m}=\int_{Z_{k}^{\hat{n},m}}\int_{X_{k}}f_{k}\left(\left.x_{k}^{\left(t\right)},Z_{k}^{m}\right|Z_{1:k-1},\left|X_{k}\right|=n\right)dx_{k}^{\left(t\right)}dz_{k}^{\left(1\right)}\cdots dz_{k}^{\left(m\right)}$$

From Equations (A21), (A25) and (A30), it can be easily seen that:

(A30) $$\omega_{k}^{\left(t\right),\hat{n},n,m}=\omega_{k}^{\hat{n},n,m}$$

Finally, by substituting Equation (A28) into Equation (A27) and then Equation (A24), we have Equation (17). This completes the proof.  ☐

## Appendix B

**Proof of Theorem 2.** The proof of Theorem 2 is similar to that of Theorem 1. Therefore, only the main differences between them are presented next. According to Assumption A.2, it follows that the predicted multi-target density $f_{k|k-1}\left(\left.X_{k}^{n}\right|Z_{1:k-1}\right)$ at time *k* is a Poisson density with intensity $\upsilon_{k|k-1}\left(x_{k}\right)$,

(A31) $$f_{k|k-1}\left(\left.X_{k}^{n}\right|Z_{1:k-1}\right)=e^{-\eta_{k|k-1}}\prod_{t=1}^{n}\upsilon_{k|k-1}\left(x_{k}^{\left(t\right)}\right)$$

where:

(A32) $$\eta_{k|k-1}=\int \upsilon_{k|k-1}\left(x_{k}\right)dx_{k}$$

denotes the expected number of predicted targets at time *k*. Substituting Equations (A32) and (13) into Equations (A2) and (A3), respectively, and integrating out $x_{k}^{\left(1\right)},\ldots ,x_{k}^{\left(n\right)}$ in the two equations, the probabilities $P_{k}\left(\left.\left|X_{k}\right|=n\right|Z_{1:k-1}\right)$ and $P_{k}\left(\left.Z_{k}^{m}\right|\left|X_{k}\right|=n\right)$ become:

(A33) $$P_{k}\left(\left.\left|X_{k}\right|=n\right|Z_{1:k-1}\right)=e^{-\eta_{k|k-1}}\eta_{k|k-1}^{n}$$

(A34) $$P_{k}\left(\left.Z_{k}^{m}\right|\left|X_{k}\right|=n\right)=e^{-\eta_{k|k-1}-\lambda_{k}}\kappa_{k}^{Z_{k}^{m}}\sum_{\theta_{n,m}}D_{k}\left(z_{k}^{\left(\theta_{n,m}\left(1\right)\right)},\ldots ,z_{k}^{\left(\theta_{n,m}\left(n\right)\right)}\right)$$

where:

(A35) $$D_{k}\left(z_{k}^{\left(\theta_{n,m}\left(1\right)\right)},\ldots ,z_{k}^{\left(\theta_{n,m}\left(n\right)\right)}\right)=\prod_{t=1}^{n}H_{k}\left(z_{k}^{\left(\theta_{n,m}\left(t\right)\right)}\right)$$

(A36) $$H_{k}\left(z_{k}^{\left(\theta_{n,m}\left(t\right)\right)}\right)=\int_{X_{k}}\upsilon_{k|k-1}\left(x_{k}^{\left(t\right)}\right)G_{k}\left(\left.z_{k}^{\left(\theta_{n,m}\left(t\right)\right)}\right|x_{k}^{\left(t\right)}\right)dx_{k}^{\left(t\right)}$$

The posterior probability $P_{k}\left(\left.\left|X_{k}\right|=n\right|Z_{1:k-1},Z_{k}^{m}\right)$ can be obtained by substituting Equations (A34) and (A35) into Equation (A1), and hence, the function $\xi_{k}^{n}\left(\left.Z_{k}^{m}\right|Z_{1:k-1}\right)$ involved in the MAP detector of Equation (A12) becomes:

(A37) $$\xi_{k}^{n}\left(\left.Z_{k}^{m}\right|Z_{1:k-1}\right)={\left(\eta_{k|k-1}\right)}^{n}\cdot \sum_{\theta_{n,m}}D_{k}\left(z_{k}^{\left(\theta_{n,m}\left(1\right)\right)},\ldots ,z_{k}^{\left(\theta_{n,m}\left(n\right)\right)}\right)$$

Substituting Equations (A32) and (13) into Equations (A15) and (A16), respectively, and integrating out $z_{k}^{\left(1\right)},\ldots ,z_{k}^{\left(m\right)}$ in Equation (A16), the density $f_{k}\left(\left.X_{k}^{n},Z_{k}^{m}\right|Z_{1:k-1}\right)$ becomes:

(A38) $$f_{k}\left(\left.X_{k}^{n},Z_{k}^{m}\right|Z_{1:k-1}\right)=\frac{e^{-\eta_{k|k-1}-\lambda_{k}}\kappa_{k}^{Z_{k}^{m}}}{\Omega_{k}^{n,m}}\sum_{\theta_{n,m}}\prod_{t=1}^{n}\upsilon_{k|k-1}\left(x_{k}^{\left(t\right)}\right)G_{k}\left(\left.z_{k}^{\left(\theta_{n,m}\left(t\right)\right)}\right|x_{k}^{\left(t\right)}\right)$$

where the normalization factor $\Omega_{k}^{n,m}$ is:

(A39) $$\Omega_{k}^{n,m}=e^{-\eta_{k|k-1}-\lambda_{k}}\lambda_{k}^{m}\sum_{\theta_{n,m}}\prod_{t=1}^{n}K_{k|k-1}^{\left(t\right)}$$

with:

(A40) Kk|k−1(t)=∫XkpD,kxk(t)υk|k−1xk(t)dxk(t)∫XkpD,kxk(t)υk|k−1xk(t)dxk(t)λkλk,θn,m(t)>0∫Xk1−pD,kxk(t)υk|k−1xk(t)dxk(t),θn,m(t)=0

Replacing $f_{k}\left(\left.X_{k}^{n},Z_{k}^{m}\right|Z_{1:k-1}\right)$ in Equation (A21) with Equation (A39), and integrating out $x_{k}^{\left(1\right)},\ldots ,x_{k}^{\left(n\right)}$ in Equation (A21), $\omega_{k}^{\hat{n},n,m}$ becomes:

(A41) $$\omega_{k}^{\hat{n},n,m}=\frac{e^{-\eta_{k|k-1}-\lambda_{k}}}{\Omega_{k}^{n,m}}\sum_{\theta_{n,m}}\int_{Z_{k}^{\hat{n},m}}\kappa_{k}^{Z_{k}^{m}}D_{k}\left(z_{k}^{\left(\theta_{n,m}\left(1\right)\right)},\ldots ,z_{k}^{\left(\theta_{n,m}\left(n\right)\right)}\right)dz_{k}^{\left(1\right)}\cdots dz_{k}^{\left(m\right)}$$

Similarly, replacing $f_{k}\left(\left.X_{k}^{n},Z_{k}^{m}\right|Z_{1:k-1}\right)$ in Equation (A25) with Equation (A39), and integrating out $x_{k}^{\left(1\right)},\ldots ,x_{k}^{\left(t-1\right)},x_{k}^{\left(t+1\right)},\ldots ,x_{k}^{\left(n\right)}$ in Equation (A25), the density $f_{k}\left(\left.x_{k}^{\left(t\right)},Z_{k}^{m}\right|Z_{1:k-1},\left|X_{k}\right|=n\right)$ becomes:

(A42) $$\begin{array}{c} f_{k}\left(\left.x_{k}^{\left(t\right)},Z_{k}^{m}\right|Z_{1:k-1},\left|X_{k}\right|=n\right)=\\ \frac{e^{-\eta_{k|k-1}-\lambda_{k}}\kappa_{k}^{Z_{k}^{m}}}{\Omega_{k}^{n,m}}\sum_{\theta_{n,m}}\frac{D_{k}\left(z_{k}^{\left(\theta_{n,m}\left(1\right)\right)},\ldots ,z_{k}^{\left(\theta_{n,m}\left(n\right)\right)}\right)}{H_{k}\left(z_{k}^{\left(\theta_{n,m}\left(t\right)\right)}\right)}\upsilon_{k|k-1}\left(x_{k}^{\left(t\right)}\right)G_{k}\left(\left.z_{k}^{\left(\theta_{n,m}\left(t\right)\right)}\right|x_{k}^{\left(t\right)}\right) \end{array}$$

The rest of the proof of Theorem 2 is completely the same as that of Theorem 1. This completes the proof.  ☐
